# Dispersal of Group A Streptococcal Biofilms by the Cysteine Protease SpeB Leads to Increased Disease Severity in a Murine Model

**DOI:** 10.1371/journal.pone.0018984

**Published:** 2011-04-25

**Authors:** Kristie L. Connolly, Amity L. Roberts, Robert C. Holder, Sean D. Reid

**Affiliations:** Department of Microbiology and Immunology, Wake Forest University School of Medicine, Winston-Salem, North Carolina, United States of America; National Institute of Allergy and Infectious Diseases, National Institutes of Health, United States of America

## Abstract

Group A *Streptococcus* (GAS) is a Gram-positive human pathogen best known for causing pharyngeal and mild skin infections. However, in the 1980's there was an increase in severe GAS infections including cellulitis and deeper tissue infections like necrotizing fasciitis. Particularly striking about this elevation in the incidence of severe disease was that those most often affected were previously healthy individuals. Several groups have shown that changes in gene content or regulation, as with proteases, may contribute to severe disease; yet strains harboring these proteases continue to cause mild disease as well. We and others have shown that group A streptococci (MGAS5005) reside within biofilms both *in vitro* and *in vivo*. That is to say that the organism colonizes a host surface and forms a 3-dimensional community encased in a protective matrix of extracellular protein, DNA and polysaccharide(s). However, the mechanism of assembly or dispersal of these structures is unclear, as is the relationship of these structures to disease outcome. Recently we reported that allelic replacement of the streptococcal regulator *srv* resulted in constitutive production of the streptococcal cysteine protease SpeB. We further showed that the constitutive production of SpeB significantly decreased MGAS5005Δ*srv* biofilm formation *in vitro*. Here we show that mice infected with MGAS5005Δ*srv* had significantly larger lesion development than wild-type infected animals. Histopathology, Gram-staining and immunofluorescence link the increased lesion development with lack of disease containment, lack of biofilm formation, and readily detectable levels of SpeB in the tissue. Treatment of MGAS5005Δ*srv* infected lesions with a chemical inhibitor of SpeB significantly reduced lesion formation and disease spread to wild-type levels. Furthermore, inactivation of *speB* in the MGAS5005Δ*srv* background reduced lesion formation to wild-type levels. Taken together, these data suggest a mechanism by which GAS disease may transition from mild to severe through the Srv mediated dispersal of GAS biofilms.

## Introduction

Cellulitis is a soft tissue infection of the dermis that extends into subcutaneous tissues and can be either non-necrotizing or more severe and associated with tissue necrosis (abscesses or exudates) [1]–[3]. This acute spreading infection can arise from a pre-existing infection, an underlying skin condition (eczema) or a break in the epithelium, and can occur at any site on the body [2]–[4]. Group A *Streptococcus* (GAS) is a Gram-positive human pathogen that is capable of causing a variety of infections in the human host, and is often associated with cellulitis and other soft tissue infections ranging in severity from impetigo to severe necrotizing fasciitis [5]–[11]. Serotype M1 GAS strains have become the most common cause of invasive GAS infections following their sudden increase in frequency and disease severity in the mid-1980's [12]. Non-invasive GAS infections, comprised of mostly throat and skin infections, are less severe but have a higher rate of occurence, with over ten million cases diagnosed each year [13].

While GAS biofilms have been observed both *in vivo* and *in vitro*, the composition and regulation of these structures during a soft tissue infection have not been well defined [14]–[17]. Akiyama *et al.* (2003) made some of the first observations of GAS microcolony formation in murine tissue infections, in which the microcolony appeared to be surrounded by glycocalyx. Similar structures were also identified in human impetigo specimens, suggesting that GAS biofilms play an important role in soft tissue pathogenesis and, subsequently, treatment of these infections [14]. *In vitro* grown biofilms have shown that DNA and proteins, not carbohydrates, are necessary components for biofilm formation, suggesting that the composition of these structures may vary between strains or in the presence of an active immune response [16].

One GAS virulence factor, SpeB, is an extracellular cysteine protease capable of cleaving both host and bacterial proteins and contributing to tissue damage and dissemination [17]–[20]. *In vitro*, SpeB has been shown to play a role in GAS evasion of the host immune response by preventing immunoglobulin and C3b, a component of the complement pathway, opsonization [21], [22]. Clearance of GAS by neutrophils and macrophages may also be inhibited in the presence of SpeB; it has been previously shown *in vitro* that SpeB can induce apoptosis in both of these phagocytic immune cells [23], [24]. SpeB activates host proteins through cleavage, such as interleukin-1β precursor and pro-matrix metalloprotease-2 and -9; these mature forms are capable of aiding in GAS dissemination from the site of infection through increased inflammation and tissue damage, respectively [17], [19]. The cleavage and degradation of extracellular matrix proteins by SpeB, such as tissue integrity components fibronectin and vitronectin, also contributes to tissue damage and bacterial colonization [18]. In addition to host proteins, SpeB degrades a wide variety of GAS-produced proteins and virulence factors, including M protein, protein F1, C5a peptidase, protein H and SmeZ [18]. SpeB cleavage of the adherence factors M protein and protein F1 is thought to reduce both bacterial and host cell-to-cell interactions [25]–[27]. Cleavage-activated C5a peptidase degrades C5a while free protein H binds C3, inhibiting opsonization by the complement pathway [26]. Finally, proteolysis of the superantigen SmeZ limits the immune response [18].

Recently, we have shown that allelic replacement of the streptococcal regulator of virulence (Srv), a putative transcriptional regulator, resulted in the constitutive production of SpeB [28], [29]. While *speB* is highly conserved and present in almost all strains of GAS, *speB* expression is variable between strains [30]. Production of SpeB in MGAS5005 planktonic culture is detected during late exponential and early stationary phases of growth, however, high levels of SpeB are present in MGAS5005Δ*srv* culture after only two hours of growth [29], [31].

Interestingly, loss of Srv also led to a significant reduction in the ability of GAS to form biofilms. As hypothesized by Donlan and Costerton, a biofilm is a bacterial sessile community encased in a matrix of extracellular polymeric substances and attached to a substratum or interface [32]. Biofilms are believed to be inherently tolerant to host defenses and antibiotic therapies and often linked to chronic illness due to impaired clearance [33], [34]. Some estimates suggest that upwards of 60% of all bacterial infections involve biofilms, including soft tissue infection and necrotizing fasciitis [14], [32], [35]. There is a growing understanding of the importance of biofilm formation in GAS disease as well [14]–[16], [36]–[40]. In our *in vitro* work we have shown that either allelic replacement of SpeB in the MGAS5005Δ*srv* background or chemical inhibition of SpeB with the cysteine protease inhibitor E64 restored biofilm formation by the MGAS5005Δ*srv* strain to wild-type levels [16], [41].

Taken together, these observations suggest two possible hypotheses for the fate of the MGAS5005Δ*srv* strain in a murine model of soft tissue infection. One, the loss of biofilm formation by MGAS5005Δ*srv in vitro* would translate into increased clearance *in vivo* and decreased virulence. Two, the constitutive production of SpeB by MGAS5005Δ*srv* would lead to increased virulence and tissue damage. To test these hypotheses, we challenged mice in a subcutaneous model of skin infection. We demonstrated that allelic replacement of *srv* resulted in increased virulence. This increased virulence was associated with increased SpeB detection and decreased evidence of MGAS5005Δ*srv* biofilm formation. Allelic replacement of *speB* in the MGAS5005Δ*srv* background reduced virulence to wild-type levels and evidence of biofilm formation *in vivo* was observed. Furthermore, local treatment of the infection with the cysteine protease inhibitor E64 significantly reduced virulence.

## Results

### Allelic replacement of *srv* resulted in increased virulence in a murine subcutaneous infection model

**Figure 1:**
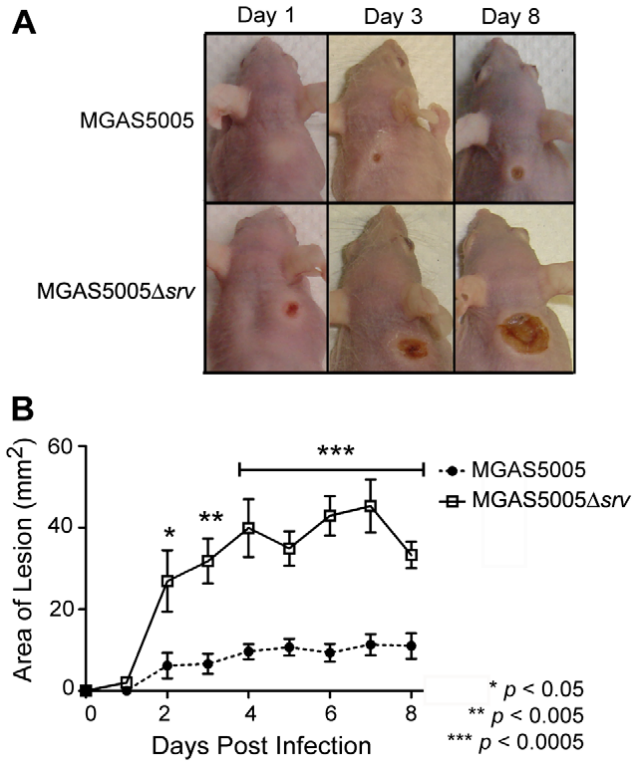
Allelic replacement of *srv* lead to increased lesion size in a murine subcutaneous infection model. (A) Groups of 10 mice (Crl:SKH1-hrBR) were challenged subcutaneously with ∼2.0×10^8^ CFU (0.1 ml) of either MGAS5005 or MGAS5005Δ*srv*. Representative images of lesions formed at 1, 3 and 8 dpi are shown. (B) The area of the lesion formed (mm^2^) was measured with a caliper daily. Lesions formed by MGAS5005Δ*srv* were significantly larger (*p*≤0.05) than those formed by MGAS5005 by 2 dpi (Student's t-test).

**Figure 2:**
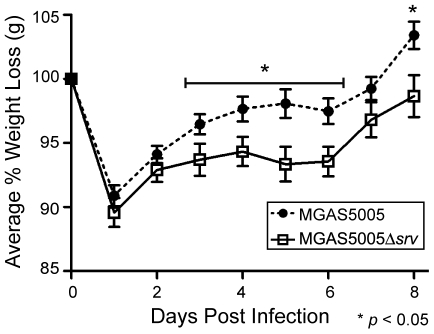
Average percentage of mouse weight loss following GAS infection. Groups of 10 mice were challenged subcutaneously with ∼2.0×10^8^ CFU (0.1 ml) of either MGAS5005 or MGAS5005Δ*srv*. The percentage of weight lost was monitored for 8 dpi. Mice infected with MGAS5005Δ*srv* weighed significantly less on 5/8 dpi (*p≤0.05).

**Figure 3:**
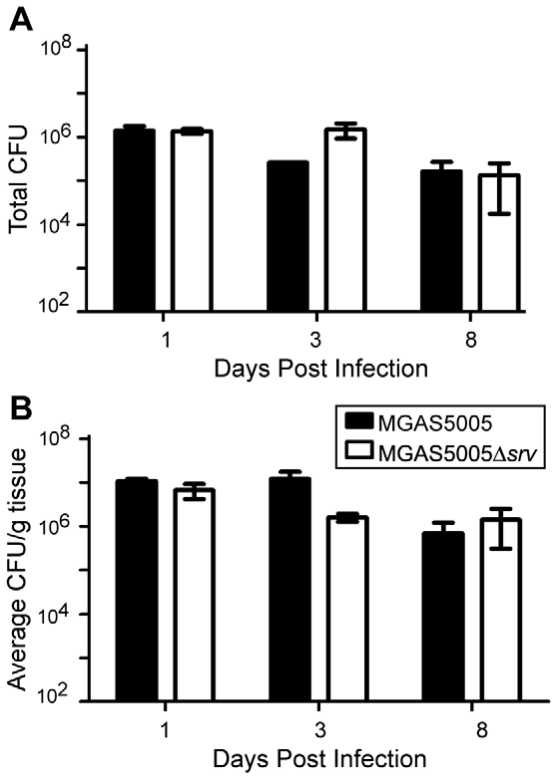
Bacterial load recovered from excised lesions. Lesions from mice infected with either MGAS5005 or MGAS5005Δ*srv* (*n* = 3 mice/strain) were excised at 1, 3 and 8 dpi, weighed and homogenized for replicate plating. No significant difference in (A) total CFU recovered or (B) CFU/g was observed at 1, 3, and 8 dpi.

To assess the loss of *srv* in an *in vivo* infection model, groups of 10 mice were inoculated with ∼2×10^8^ CFU of either MGAS5005 or MGAS5005Δ*srv*. The area of the lesion, average percentage of weight loss, and bacterial load recovered were recorded. In general, MGAS5005 infected animals developed a subcutaneous abscess 1 dpi that erupted as a measurable cutaneous lesion by 2 dpi (Figure 1A). In contrast, MGAS5005Δ*srv* infected animals developed readily visible lesions by 2 dpi that were significantly larger than those observed in MGAS5005 infected animals (Figure 1A and B). This trend continued with significantly larger lesions observed in MGAS5005Δ*srv* infected animals throughout the course of the experiment with lesions exceeding 40 mm^2^ in some cases (Figure 1A and B). A similar trend was observed when the average weight lost between the two groups of animals was compared. Both groups of animals lost on average 10% of their body weight by 1 dpi (Figure 2). By day 3, MGAS5005Δ*srv* infected animals weighed significantly less than their wild-type infected counterparts (Figure 2). While not always significant, the average recorded weight of MGAS5005Δ*srv* infected animals was less than MGAS5005 infected animals over the 8 day experiment (Figure 2). To determine if this increase in virulence might be due to increased bacterial load, three additional mice for each experimental group were infected as described above. Lesions and the underlying abscess were surgically excised, homogenized, and the bacteria were enumerated. Even though larger lesions were excised from MGAS5005Δ*srv* infected animals, the total bacterial CFU recovered (Figure 3A), as well as bacterial CFU/g (Figure 3B), was not statistically different between MGAS5005 and MGAS5005Δ*srv* infected animals at 1, 3, or 8 dpi.

### Histopathology of lesion tissue sections revealed greater necrosis in MGAS5005Δ*srv* infected samples

**Figure 4:**
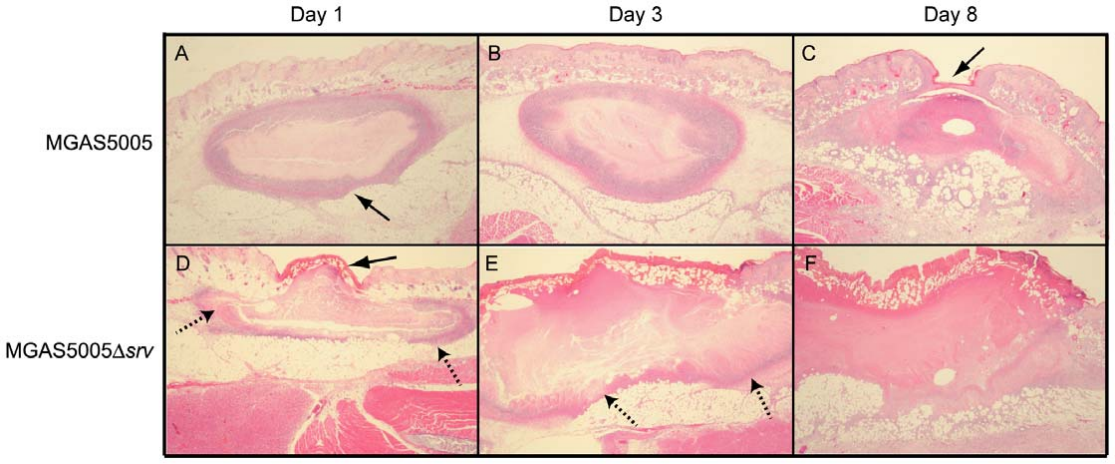
Histopathology of excised lesions from MGAS5005 and MGAS5005Δ*srv* infections. Lesions were surgically excised at days 1, 3, and 8 post infection. 10 µm sections were subjected to H&E staining. Representative low-magnification images (2×) from each time point are shown. (A,B) Infection with MGAS5005 resulted in the formation of a subcutaneous abscess (arrow) that was well delineated by fibrin (pink border) and PMNs (purple border). (C) By 8 dpi, the abscess had ruptured and formed a cutaneous lesion that showed signs of healing (arrow). (D) MGAS5005Δ*srv* infection resulted in a cutaneous lesion (arrow). (D & E) Note the subcutaneous abscess was less contained by colocalized fibrin and PMNs (dashed arrows). (E) The cutaneous lesion grew in size and did not show any appreciable healing by 8 dpi (F).

To further investigate the differences in virulence observed, 3 mice per experimental group were infected as before and histopathology was performed using sections from MGAS5005 and MGAS5005Δ*srv* lesions collected at days 1, 3, and 8 post-infection. 10 µm sections were subjected to H&E staining to observe the infiltrate present and the extent of damage at the site of infection (Figure 4). MGAS5005 infected samples showed the clear development of a subcutaneous abscess, characterized by edema well delineated by fibrin and polymorphonuclear leukocytes (PMNs) (Figure 4A and B). While the extent of ulceration varied, cutaneous lesions developed over the next three days with some degree of epithelial reformation (healing) observed by 8 dpi (Figure 4C). In contrast, MGAS5005Δ*srv* infected samples showed evidence of edema and ulceration by 1 dpi, however, the edema was not well delineated by fibrin accumulation and PMN influx (Figure 4D). As recorded photographically in Figure 1, the MGAS5005Δ*srv* lesions continued to develop over time with little signs of healing or resolution (Figure 4 E and F).

### SpeB detected throughout MGAS5005Δ*srv* infected tissue

**Figure 5:**
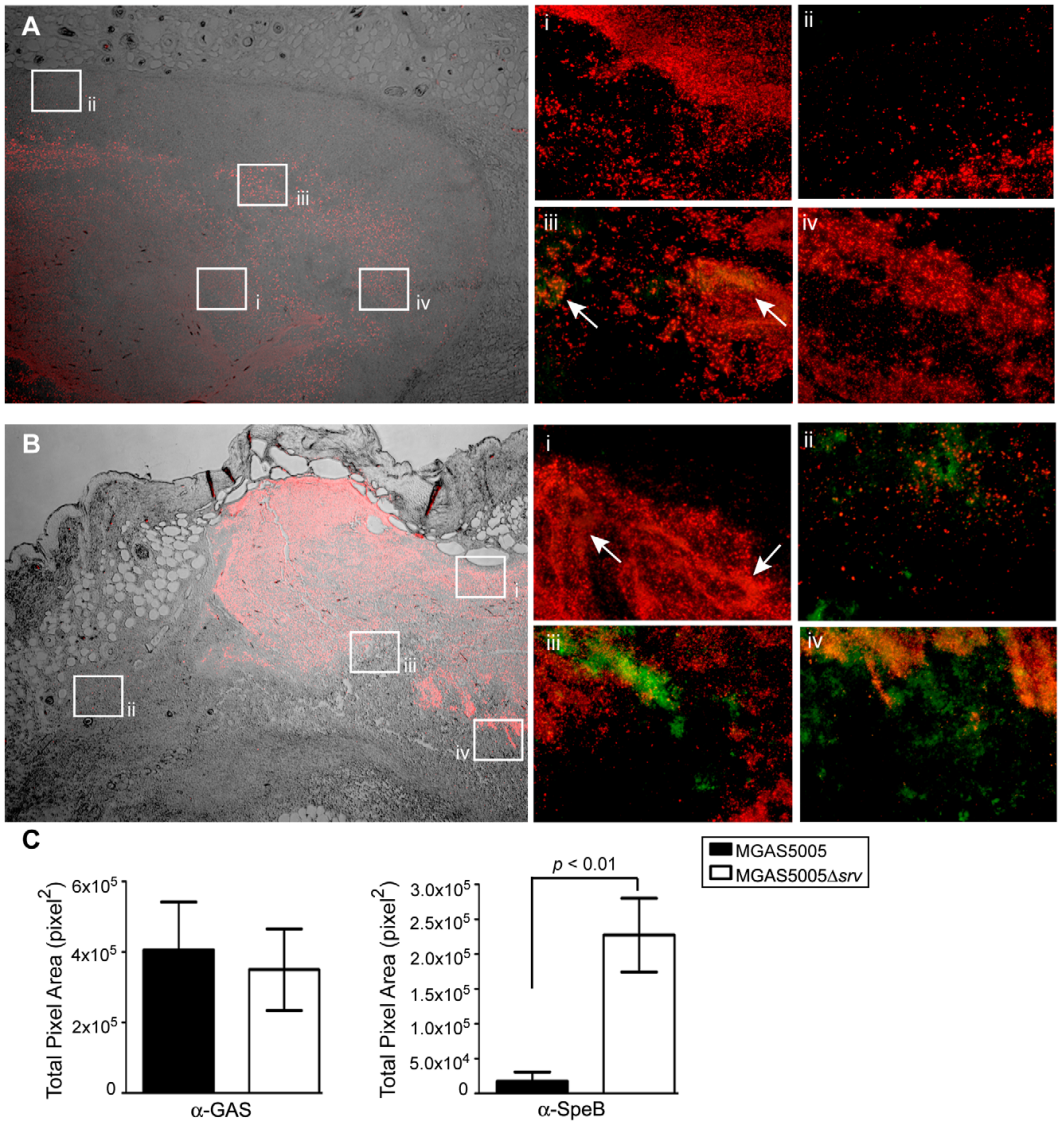
Immunofluorescent antibody staining revealed detectable levels of SpeB throughout MGAS5005Δ*srv* infected tissue as compared to MGAS5005 infected tissue. Subcutaneous abscesses from (A) MGAS5005 and (B) MGAS5005Δ*srv* infections were excised 1 dpi, sectioned, and stained with rabbit anti-SpeB sera and goat anti-GAS sera, and the appropriate fluorescent secondary antibody conjugate. (A, B) DIC/fluorescent images (4×) from an MGAS5005 infected animal (A) and an MGAS5005Δ*srv* infected animal (B) show the distribution of GAS (red) throughout the abscess. Randomly selected areas throughout the abscesses were examined for the colocalization of GAS and SpeB (20×, i–iv). MGAS5005 was readily detected (Ai–iv), but SpeB (green) was rarely detected in MGAS5005 infected samples (arrows, Aiii). In contrast, SpeB was detected in the presence of MGAS5005Δ*srv* throughout the infected samples (Bi–iv). Colocalized SpeB and GAS appear yellow. Representative images are shown. (C) Average total area of pixels (pixels^2^) was calculated for anti-GAS and anti-SpeB staining in the representative images shown of MGAS5005 and MGAS5005Δ*srv*. Comparable amounts of anti-GAS staining was observed, however, there is significantly more anti-SpeB staining in MGAS5005Δ*srv* images compared to MGAS5005 (* *p*<0.01).

As mentioned previously, our *in vitro* work demonstrated that allelic replacement of *srv* resulted in constitutive production of SpeB. To begin to test the hypothesis that the increased virulence observed of MGAS5005Δ*srv* was due to SpeB, we used immunofluorescent microscopy to look for the presence of GAS and SpeB in the infected tissue. Given that a significant difference in lesion size was observed by 2 dpi, we elected to study tissue samples collected 1 dpi. Adjacent 10 µm sections of lesion tissue to those collected for histology and Gram-stain were obtained and stained with rabbit anti-SpeB sera, goat anti-GAS sera, and fluorescent secondary antibody conjugates. DIC/fluorescent images showed GAS distributed throughout MGAS5005 and MGAS5005Δ*srv* infected samples (Figure 5A and B). Randomly selected areas throughout the abscesses were chosen for closer examination at 20× magnification (Figure 5A and B i–iv). While MGAS5005 was readily detected in the 20× images, SpeB was rarely observed (Figure 5Ai–iv). However, SpeB was readily detected in the MGAS5005Δ*srv* infected samples (Figure 5Bi–iv). In an effort to quantify the signal observed, ImageJ (rsbweb.nih.gov) was used to calculate the pixel area from the four representative 20× images provided (Figure 5C). While the images shown are from single mouse infections, they are representative of the images obtained from each of the experimental groups. While both infections showed similar staining of anti-GAS, anti-SpeB staining was significantly increased in images collected from the MGAS5005Δ*srv* infected tissue sample (Figure 5C).

### Gram-staining revealed microcolonies indicative of biofilms in MGAS5005 infected samples

**Figure 6:**
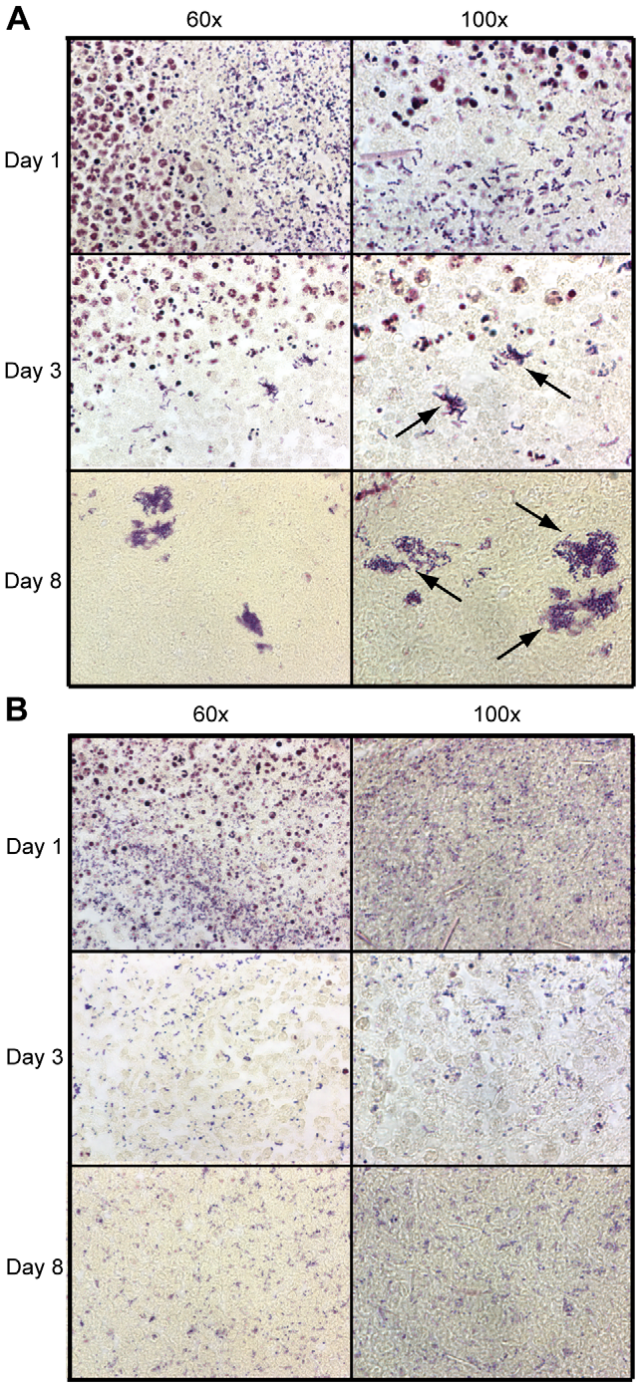
Gram-staining of lesion tissue sections revealed the presence of MGAS5005 microcolonies (biofilms). 10 µm sections of lesion tissue collected 1, 3, and 8 dpi were subjected to Gram-staining. (A) MGAS5005 infected samples contained microcolonies of adherent GAS which were visible by 3 dpi (arrows). These microcolonies are reminiscent of biofilms and appeared to increase in size by 8 dpi. (B) MGAS5005Δ*srv* infected samples contained randomly dispersed GAS throughout the field of view. Microcolonies were largely absent. The same view of single day images are shown at 60× and 100× magnification. Representative images are shown.

Based on our *in vitro* data, we hypothesized that MGAS5005Δ*srv* would be largely unable to form biofilms *in vivo*. We and other researchers have shown that microcolonies, detected by Gram-staining and other methods, are evidence of *in vivo* biofilms [14], [15], [17], [42]. 10 µm sections of lesion tissue collected 1, 3, and 8 dpi were subjected to Gram-staining. MGAS5005 microcolonies were clearly observed by 3 dpi (Figure 6A). Larger microcolony formations were observed 8 dpi in MGAS5005 infected samples (Figure 6A). However, no comparable structures were observed in Gram-stained MGAS5005Δ*srv* infected samples (Figure 6B). Instead, MGAS5005Δ*srv* appeared randomly distributed throughout the samples.

### Allelic replacement of s*peB* in the MGAS5005Δ*srv* background significantly reduced lesion formation in infected animals and restored microcolony formation

**Figure 7:**
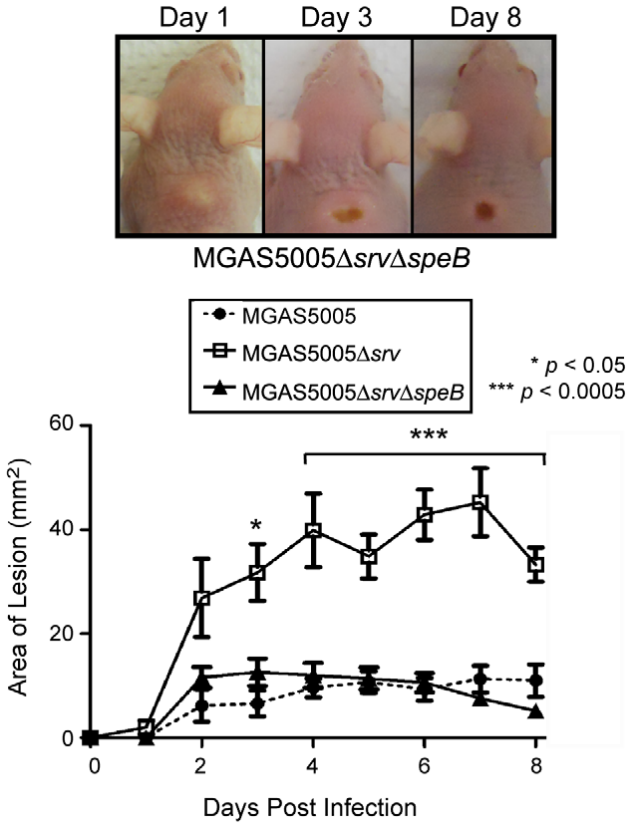
Allelic replacement of *speB* in the MGAS5005Δ*srv* background resulted in significantly decreased lesion size. Representative images of lesions formed in mice at 1, 3 and 8 days following subcutaneous infection with ∼2×10^8^ CFU (0.1 ml) of MGAS5005Δ*srv*Δ*speB*. Lesion development (mm^2^) was monitored over 8 days using a caliper (*n* = 10 mice/strain). A significant reduction in lesion size was observed in MGAS5005Δ*srv*Δ*speB* infected mice (*p*<0.05). The size of lesions observed in MGAS5005 infected mice vs. MGAS5005Δ*srv*Δ*speB* was not significantly different.

**Figure 8:**
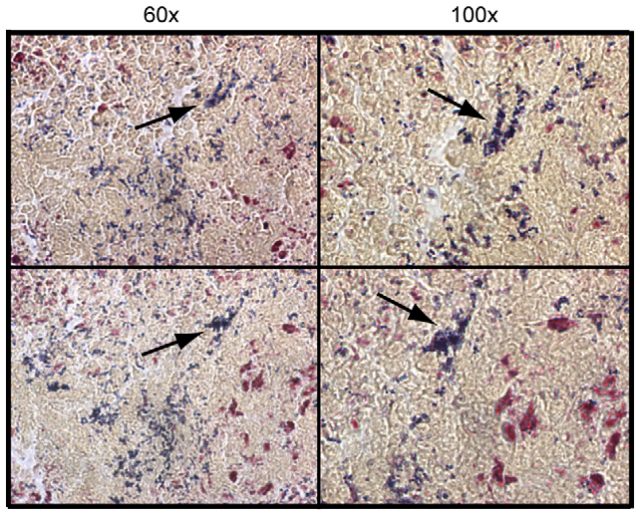
Microcolony formation is observed in MGAS5005Δ*srv*Δ*speB* infected tissue. Representative images of Gram-stained sections (10 µm thick) collected from two MGAS5005Δ*srv*Δ*speB* infected mice at 8 dpi. MGAS5005Δ*srv*Δ*speB* microcolonies (arrows) were present in the edema at the site of infection.

We have recently shown that allelic replacement of *speB* in the MGAS5005Δ*srv* background restored biofilm formation and eliminated SpeB production in the MGAS5005Δ*srv* strain [41], [42]. Based on the data presented here we hypothesize that the MGAS5005Δ*srv*Δ*speB* strain would be less virulent and microcolony formation in the infected lesion tissue samples would be observed. Mice were infected as before using ∼2×10^8^ CFU of MGAS5005Δ*srv*Δ*speB* (*n* = 10). Lesion formation by the MGAS5005Δ*srv*Δ*speB* strain resembled that of MGAS5005 and was significantly less than the size of lesions observed in MGAS5005Δ*srv* infected samples (Figure 7). Comparable bacterial CFU was recovered at 8 dpi from MGAS5005, MGAS5005Δ*srv*, and MGAS5005Δ*srv*Δ*speB* infected tissue (data not shown). Gram-stained 10 µm sections of MGAS5005Δ*srv*Δ*speB* infected samples revealed the presence of microcolonies of varying sizes 8 dpi (Figure 8).

### Chemical inhibition of SpeB *in vivo* during MGAS5005Δ*srv* infection significantly reduces lesion formation

**Figure 9:**
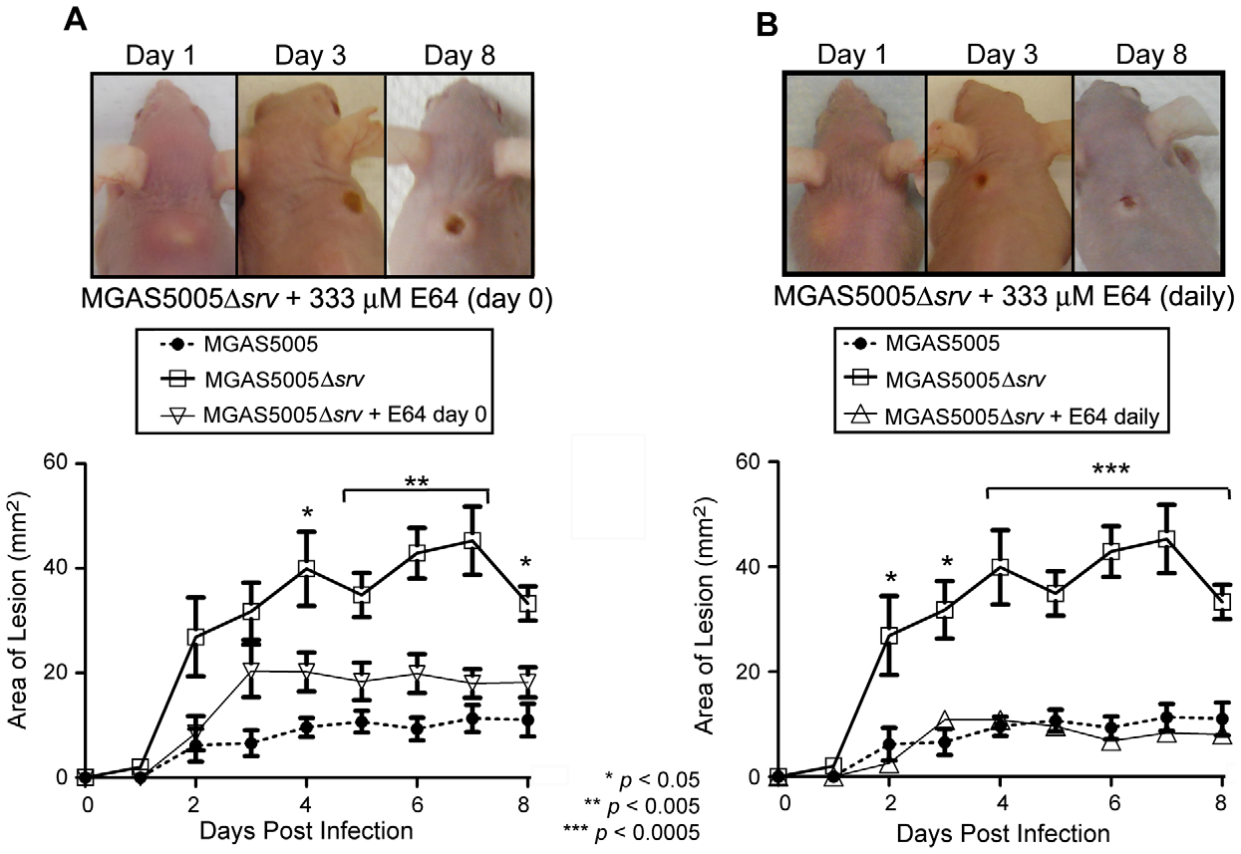
Use of the chemical inhibitor of cysteine proteases E64 significantly reduced lesion size in MGAS5005Δ*srv* infected animals. Representative images of lesions formed in mice at 1, 3 and 8 days following subcutaneous infection with ∼2×10^8^ CFU of MGAS5005Δ*srv*. (A) The infecting dose of MGAS5005Δ*srv* was suspended in 333 uM E64 (0.1 ml), and lesion development (mm^2^) was monitored over 8 days (*n* = 10 mice). A significant reduction in lesion formation was observed when E64 was inoculated with the infecting dose of MGAS5005Δ*srv* compared to inoculation with MGAS5005Δ*srv* alone (*p*<0.05). (B) Following inoculation of animals with E64+MGAS5005Δ*srv* as before, an additional inoculation of 333 µM E64 (0.1 mL) was injected directly into the abscess each day following infection (*n* = 10). A significant reduction in lesion size was observed with E64 treated animals forming lesions roughly equivalent in size to untreated MGAS5005 infected animals.

Our data indicate that increased virulence of the MGAS5005Δ*srv* strain is due to the constitutive production of SpeB documented *in vitro*. Thus, we hypothesized that chemical inhibition of SpeB *in vivo* during infection would reduce lesion formation and virulence. To test this hypothesis, the infecting dose of MGAS5005Δ*srv* was suspended in 333 µM of E64 (0.1 mL). E64 is a commercially available inhibitor of cysteine proteases [43], [44]. Mice were infected as before and monitored for 8 days (*n* = 10). The overall area of the lesions formed was reduced and was significantly less than lesions formed by MGAS5005Δ*srv* infected animals on days 4–8 post-infection (Figure 9A). To determine if lesion size could be reduced even further, E64 was mixed with the inoculating dose of MGAS5005Δ*srv* as before, and infected mice received injections of 0.1 mL 333 µM E64 each day post-infection. Injections were delivered directly to the subcutaneous abscess. Lesion formation was returned to wild-type levels and significantly less than in untreated MGAS5005Δ*srv* infected animals days 2–8 post-infection (Figure 9B). Addition of E64 to MGAS5005 infected samples did not result in a statistically significant change in lesion size over the course of infection (data not shown).

**Figure 10:**
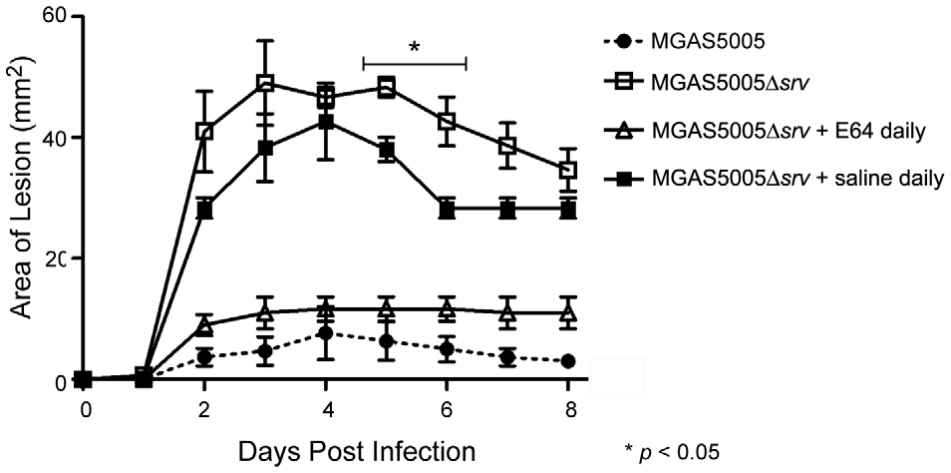
Daily wound irrigation not responsible for the reduction in lesion size observed in E64 treated animals. Lesion size in saline treated animals (*n* = 3) was significantly reduced at 5 and 6 dpi compared to MGAS5005Δ*srv* infected animals (*p*<0.05), however, lesions in saline treated animals were statistically larger than those in E64 treated animals at 2–8 dpi (*p*<0.05).

To rule out the possibility that the reduced lesion size was due to the therapeutic effects of lavage, animals were infected MGAS5005Δ*srv* as before and receive daily 0.1 mL injections of saline directly to the abscess. While lesion size was reduced, it was not reduced to the extent of the E64 treated animals (Figure 10). Thus, while lavage has a therapeutic effect, chemical inhibition of SpeB by E64 *in vivo* significantly reduces the virulence of MGAS5005Δ*srv*.

## Discussion

Previously, we have shown that the loss of the stand-alone response regulator Srv in MGAS5005 resulted in significant reduction of *in vitro* biofilm formation in both static and flow biofilm assays [16], [41]. Furthermore, MGAS5005Δ*srv* exhibited reduced biofilm formation *in vivo* in a chinchilla model of otitis media [42]. The loss of biofilm formation by MGAS5005Δ*srv* was attributed to constitutive production of the cysteine protease SpeB, as biofilm formation was restored through either chemical inhibition of SpeB *in vitro* or allelic replacement of *speB* in the MGAS5005Δ*srv* background in both *in vitro* and *in vivo* biofilm models [16], [41], [42]. One long term goal of our laboratory is to understand the role of the GAS biofilm in disease. In our recent work utilizing a chinchilla model of otitis media, we hypothesized that the biofilm deficient MGAS5005Δ*srv* strain would be readily cleared from the site of infection due to the lack of the protective properties afforded by the biofilm. However, we found that MGAS5005Δ*srv* persisted at the site of infection for the duration of the experiment [42]. Furthermore, higher bacterial loads of MGAS5005Δ*srv* were observed in middle ear effusions throughout the course of infection compared to MGAS5005, and, while not statistically significant, the mortality rate of MGAS5005Δ*srv* infected animals was higher [42]. Taken together, this data supports a hypothesis that dispersal of the GAS biofilm by SpeB results in increased virulence. Here, we chose to further explore this hypothesis in a murine soft tissue model of infection. Based on our hypothesis, one of two outcomes was likely. One, lack of biofilm formation in the soft tissue model would result in the accelerated clearance of GAS from the site of infection. Alternatively, dispersal of the GAS biofilm and the constitutive production of SpeB would result in increased virulence and tissue damage. The results presented here clearly support the latter outcome.

The association of SpeB and increased lesion development has been previously reported in other GAS infection models [10], [20], [45]–[48]. For example, SpeB production was found to be essential for establishing a murine skin infection that ultimately resulted in systemic infection, reduced clearance by the innate immune response, and increased mortality [10], [20]. More recently, SpeB levels positively correlated with the severity of tissue damage observed following a GAS skin infection in a humanized mouse model [49]. Furthermore, addition of purified SpeB with either a wild-type or *ΔspeB* M1 strain into a mouse air sac model of infection led to accelerated and increased tissue necrosis, as well as dissemination of the organism away from the initial site of infection [47]. The Δ*speB* M1 strain alone did not form a lesion of any significance [47]. Thus, SpeB is a well appreciated and increasingly understood virulence factor of GAS. However, there are several observations presented here that provide new insight into the biology of GAS and its pathogenesis.

First, we clearly show that allelic replacement of *srv* in the MGAS5005 background leads to increased virulence coupled with increased production of SpeB *in vivo*. Previously, regulation of *speB* has been linked to several regulatory factors, including the two component signal transduction system CovRS (also referred to as CsrRS) [50]–[53]. This is particularly interesting because MGAS5005 has been shown to produce a truncated, functionally inactive CovS protein, which results in a loss of the histidine kinase domain and the ability to phosphorylate CovR [48], [54], [55]. It should be noted that this does not invalidate MGAS5005 as a strain worthy of study. MGAS5005 was isolated from a patient suffering from invasive disease. In fact, several recent studies have shown evidence of GAS with *covS* non-functional mutations isolated from *in vivo* systemic infections, suggesting that *covS* mutants posses a selective advantage during invasive infections [48], [54], [56]–[61]. Normally, CovS can also help to relieve CovR repression and increase *speB* expression in response to stress [62], [63]. Complementation of MGAS5005 in *trans* with a functional *covS* restored SpeB production [48]. Here, SpeB production is restored through the allelic replacement of *srv* suggesting that Srv is involved in the CovR mediated repression of *speB*. Efforts are underway to understand this mechanism as are studies to explore the function of Srv in strain backgrounds with a functional CovS.

Second, loss of *srv* also results in a loss of biofilm formation *in vivo*. This is observed in our Gram-stained sections which revealed that MGAS5005 aggregated into microcolonies while MGAS5005Δ*srv* was dispersed throughout the infected samples. This change in phenotype is also due to the restored production of SpeB as microcolony formation was observed in MGAS5005Δ*srv*Δ*speB* infected samples. These results mimic what we have recently observed in a chinchilla model of GAS otitis media [42]. Thus, we now have evidence that biofilm formation is not required for infection at two distinct host sites (skin and middle ear), or at least not required given the means of inoculation used. However, our data also suggests that MGAS5005 would naturally form a biofilm upon infection. We envision a model where biofilm formation is used for colonization of a host site and protection from the innate immune response. Coordinate regulation of *speB* by both CovR and Srv (and perhaps other regulators) would allow for the controlled production of SpeB that would facilitate dispersal of some portion of GAS from the biofilm to achieve spread to another host site or susceptible host. Under this model, loss of regulation of this system would lead to severe disease. It has been hypothesized that downregulation of SpeB in Δ*covS* strains would prevent cleavage and degradation of virulence factors that may aid in GAS transitioning from a localized to a systemic infection [45], [54], [56], [57], [64], [65]. It is interesting to speculate that the selective advantage provided by natural mutations in *covS* is an increase in the ability to form biofilms and a resulting increase in the ability to colonize a host site. We have begun to test this hypothesis and have found that allelic replacement of *srv* in a normally CovS+ background in serotypes M1, M3, M12 and M18 resulted in decreased biofilm formation *in vitro* (data not shown).

Finally, we demonstrated that chemical inhibition of SpeB *in vivo* resulted in a significant reduction in lesion formation. We utilized the specific inhibitor of cysteine proteases E64, which irreversibly binds the active thiol group of SpeB [43], [44], [66]. E64 is commonly used as a cysteine protease inhibitor during *in vitro* assays, but this is the first study to our knowledge to demonstrate the effects of E64 treatment on GAS infection *in vivo* [11], [16], [65], [67], [68]. Addition of E64 to the MGAS5005Δ*srv* inoculum immediately prior to infection significantly reduced lesion development, suggesting that halting SpeB activity early during the course of infection may be the most useful. Daily treatment of MGAS5005Δ*srv* infections with E64 further reduced lesion development. Of course E64 may be inhibiting other inflammatory elements at the site of infection which contribute to lesion development. Taken together, this data provides further support for therapeutics designed to modulate SpeB and the host immune response to streptococcal infection.

## Materials and Methods

### Ethics statement

This study was carried out in strict accordance with the recommendations in the Guide for the Care and Use of Laboratory Animals of the National Institutes of Health. The protocol was approved by the Animal Care and Usage Committee of the Wake Forest University School of Medicine (Animal Welfare Assurance #A3391-01). All procedures were performed under isoflurane anesthesia, and all efforts were made to minimize suffering.

### Bacterial strains and growth conditions

MGAS5005 is a M1T1 serotype strain isolated from a case of invasive GAS disease and has previously been used in several studies of GAS pathogenicity [16], [69]. The isogenic mutants MGAS5005Δ*srv* and MGAS5005Δ*srv*Δ*speB* were generated by allelic replacement as previously described [69]–[71]. Overnight cultures grown in Todd Hewitt broth (Becton-Dickinson) supplemented with 2% yeast extract (THY) (Fisher Scientific) at 37°C, 5% CO_2_ were diluted into fresh THY and allowed to reach logarithmic phase. Logarithmic cultures were washed 3 times in 1× Dulbecco's Phosphate Buffered Saline (DPBS) before infection. Initial CFU of infectious dose was confirmed by serial dilutions plated onto THY agar plates.

### Murine subcutaneous infections

Studies were approved by the Animal Care and Use Committee of Wake Forest University Health Sciences. Five-week-old, outbred, immunocompetent, hairless female Crl:SKH1-*hr*BR mice (Charles River) received subcutaneous injections of ∼2.0×10^8^ CFU (0.1 ml) of either MGAS5005, MGAS5005Δ*srv* or MGAS5005Δ*srv*Δ*speB* at the base of the neck. Mice that received L-trans-Epoxysuccinyl-leucylamido(4-guanidino)butane (E64) (Sigma) treatment were given ∼2.0×10^8^ CFU MGAS5005Δ*srv* resuspended in 333 µM E64 (0.1 ml) at the time of infection. Those that were given daily treatments of E64 received 333 µM E64 (0.1 ml) injected at the site of infection beginning 24 hours post infection. Area of the lesion formed at the site of infection was measured daily using a caliper. The weight of each mouse was recorded daily for up to 8 days following infection, at which point the mice were euthanized and tissue at the site of infection was excised. A random subset of lesions were homogenized to determine the bacterial load (CFU/g) at 1, 3 and 8 days following infection. Tissue samples were also fixed for paraffin embedding or snap frozen in liquid nitrogen and stored at −80°C.

### Microscopy

Tissue samples were fixed in fresh 1% paraformaldehyde for 24 hours at 4°C and then stored in 70% ethanol at room temperature until paraffin embedding. Adjacent 10 µm thick sections were collected and used for hematoxylin and eosin staining (H&E), Gram-staining, or immunofluorescence. Sections were collected on positively charged slides and heat fixed at 85°C for 10 minutes. Paraffin was removed from the tissue sections by xylene and ethanol washes before samples were stored in 1× DPBS. Sections were stained using Harris's hematoxylin formula, eosin-phloxine and a standard H&E protocol. Taylor's Brown-Brenn modified Gram-stain was used for Gram-staining tissue sections.

Immunofluorescence utilized double staining with the primary antibodies rabbit anti-SpeB (1∶100) (Toxin Technology, Inc.) and goat anti-GAS (1∶500) (US Biologicals); rabbit anti-*Borrelia burgdorferi* (1∶500) (US Biologicals) was used as a negative control. Secondary antibodies used for double staining were Alexa Fluor-488 donkey anti-rabbit (1∶500) and Alexa Fluor-568 donkey anti-goat (1∶500) (Invitrogen). Samples were blocked in 1% bovine serum albumin (BSA)-DPBS for 1 hour at room temperature. Primary antibodies in 1% BSA-DPBS were applied to the samples and incubated for 30 minutes at 37°C in a humidified chamber; these same conditions were repeated for addition of the secondary antibodies. A glass coverslip was fixed with ProLong Gold antifade reagent (Invitrogen) and prepared samples were allowed to cure overnight in the dark at room temperature. Images were captured using a Nikon Eclipse TE300 Light Microscope (Nikon) and QImaging Retiga-EXi camera (AES). ImageJ v1.43 software (rsbweb.nih.gov) was used to analyze total pixel counts and store images.
